# Noncontiguous finished genome sequence and description of *Raoultibacter massiliensis* gen. nov., sp. nov. and *Raoultibacter timonensis* sp. nov, two new bacterial species isolated from the human gut

**DOI:** 10.1002/mbo3.758

**Published:** 2019-01-30

**Authors:** Sory Ibrahima Traore, Melhem Bilen, Mamadou Beye, Awa Diop, Maxime Descartes Mbogning Fonkou, Mamadou Lamine Tall, Caroline Michelle, Muhammad Yasir, Esam Ibraheem Azhar, Fehmida Bibi, Fadi Bittar, Asif Ahmad Jiman‐Fatani, Ziad Daoud, Fréderic Cadoret, Pierre‐Edouard Fournier, Sophie Edouard

**Affiliations:** ^1^ UMR MEPHI, IRD, APHM, IHU Méditerranée‐Infection Aix‐Marseille Université Marseille France; ^2^ Clinical Microbiology Department, Faculty of Medicine and Medical sciences University of Balamand Amioun Lebanon; ^3^ UMR VITROME, IRD, AP‐HM, SSA, IHU Méditerranée‐Infection Aix‐Marseille Université Marseille France; ^4^ Special Infectious Agents Unit, King Fahd Medical Research Center King Abdulaziz University Jeddah Saudi Arabia; ^5^ Medical Laboratory Technology Department, Faculty of Applied Medical Sciences King Abdulaziz University Jeddah Saudi Arabia; ^6^ Department of Medical Microbiology and Parasitology, Faculty of Medicine King Abdulaziz University Jeddah Saudi Arabia

**Keywords:** culturomics, human gut microbiota, new bacterial species, *Raoultibacter massiliensis*, *Raoultibacter timonensis*, taxonogenomics

## Abstract

As part of the culturomics project aiming at describing the human microbiota, we report in this study the description of the new bacterial genus *Raoultibacter* gen. nov. that includes two new species, *that is*,* R. massiliensis* sp. nov. and *R. timonensis* sp. nov. The *R. massiliensis* type strain Marseille‐P2849^T^ was isolated from the fecal specimen of a healthy 19‐year‐old Saudi Bedouin, while *R. timonensis* type strain Marseille‐P3277^T^ was isolated from the feces of an 11‐year‐old pygmy female living in Congo. Strain Marseille‐P2849^T^ exhibited 91.4% 16S rRNA sequence similarity with *Gordonibacter urolithinfaciens*, its phylogenetic closest neighbor with standing in nomenclature. As well, strain Marseille‐P3277^T^ exhibited 97.96% 16S rRNA similarity with strain Marseille‐P2849^T^. Both strains were Gram‐positive, motile, nonspore‐forming rod and form transparent microcolonies on blood agar in both anaerobic and microaerophilic atmospheres. The genome sizes of strain Marseille‐P2849^T^ and strain Marseille‐P3277^T^ were 3,657,161 bp and 4,000,215 bp, respectively. Using a taxono‐genomic approach combining the phenotypic, biochemical, and genomic characteristics, we propose the genus *Raoultibacter* gen. nov., which contains strains Marseille‐P2849^T^ (= CSUR P2849^T^, = DSM 103407^T^) and Marseille‐P3277^T^ (=CCUG 70680^T^, =CSUR P3277^T^) as type strains of the species *R. massiliensis* sp. nov., and *R. timonensis* sp. nov., respectively.

## 1 INTRODUCTION

The human microbiota is a highly diverse consortium of microbes colonizing different regions of the human body. The role of the microbiota took an important interest in the scientific and medical communities as it was demonstrated to be involved in human health (Alegre, Mannon, & Mannon, 2014; Glenwright et al., 2017; Honda & Littman, 2016; Round & Mazmanian, 2009). For instance, a dysbiosis of the microbiota has been proven to be implicated in a growing number of pathologies and its modulation can have beneficial impacts on the host (Smits, Bouter, de Vos, Borody, & Nieuwdorp, 2013; Zak‐Gołąb, Olszanecka‐Glinianowicz, Kocełak, & Chudek, 2014). Over the past decade, great advances have been achieved by the development of next‐generation DNA sequencing technologies, which led to a considerable progress in the study of different ecosystems including the intestinal microbiota (Margulies et al., 2005). However, many drawbacks appeared when using these molecular methods, such as the inability to distinguish between dead or living bacteria and the depth bias that neglects a minority but important bacterial species (Greub, 2012). Consequently, a new approach “culturomics” was developed in our laboratory in order to exhaustively explore the microbial ecosystems and to increase the chance of isolating previously uncultured bacteria (Lagier et al., 2012, 2015, 2016 ). Culturomics relies on the multiplication of culture conditions (including the variation of temperature, media, atmosphere…) and is coupled by a rapid bacterial identification method, the matrix‐assisted laser desorption/ionization time‐of‐flight mass spectrometry (MALDI‐TOF‐MS). The latter proved its efficiency in describing the human gut microbiota by reporting a significant number of previously uncultured and novel bacterial species (Lagier et al., 2016). Nevertheless, we are still far from understanding the human microbiome since only around 2,776 human bacterial species have been isolated, knowing that up to 1,012 bacteria are estimated to be present in only 1 g of stool (Bilen et al., 2018; Hugon et al., 2015). In the present work, the two studied organisms, strains Marseille‐P2849^T^ and Marseille‐P3277^T^, were isolated from the stool samples of a 19‐year‐old healthy Saudi Bedouin and an 11‐year‐old Congolese pygmy female, respectively. These bacteria were not identified using MALDI‐TOF‐MS. The sequencing and phylogenetic analysis of their 16S rRNA genes classified them as members of a new genus within the family *Eggerthellaceae* (Gupta, Chen, Adeolu, & Chai, 2013). This family contains the type genus *Eggerthella* and the genera *Adlercreutzia*,* Asaccharobacter*,* Cryptobacterium*,* Denitrobacterium*,* Enterorhabdus*,* Gordonibacter*,* Paraeggerthella*,* Enteroscipio*,* Rubneribacter*, and *Slackia* (Gupta et al., 2013). Among its members, *Eggerthella lenta* is commonly detected in humans and has been associated with bacteremia in patients with intraabdominal or gastrointestinal tract pathologies, bacteremia complicated by spondylodiscitis, psoas abscess, and meningitis (Gardiner et al., 2015; Gardiner, Korman, & Junckerstorff, 2014; Wong, Aoki, & Rubinstein, 2014). We herein describe the new genus *Raoultibacter* gen. nov. within the family *Eggerthellaceae* using the taxono‐genomic approach (Fournier & Drancourt, 2015). Strain Marseille‐P2849^T^ (= CSUR P2849, = DSM 103407) is the type strain of the new species *Raoultibacter massiliensis* sp. nov and Marseille‐P3277^T^ is the type strain of the species *Raoultibacter timonensis* sp. nov (=CCUG 70680, =CSUR P3277).

## 2 METHODS AND MATERIALS

### 2.1 Ethical requirements and sample collection

Strain Marseille‐P2849^T^ was isolated in April 2016 from the stool sample of a 19‐year‐old healthy Bedouin male living in Saudi Arabia. As for strain Marseille‐P3277^T^, it was isolated in June 2016 from the stool specimen of an 11‐year‐old healthy Pygmy female living in Congo. The fecal specimens were preserved at 4°C and sent to Marseille, where they were stored at −80°C in 2015. The donors gave a signed informed consent, and the study was validated by the ethics committee of the Institut Federatif de Recherche 48 under number 09‐022.

### 2.2 Isolation of the strains

Stool samples were diluted with phosphate‐buffered saline (Life Technologies, Carlsbad, CA, USA) and multiple culture conditions were applied as previously described (Jean‐Christophe Lagier et al., 2016). Bacterial growth assessment was done by directly culturing samples from the blood culture bottles on Columbia blood agar (Biomerieux, France). Strain Marseille‐P2849^T^ was isolated after stool sample's incubation in an anaerobic blood culture bottle (Becton‐Dickinson, BACTEC Plus anaerobic/F Media, Le pont de Claix, France) supplemented with 5 ml filtered rumen for 7 days at 37°C. Similarly, strain Marseille‐P3277^T^ was isolated after 2 days of stool sample incubation in an anaerobic blood culture bottle supplemented with 5 ml sterile sheep blood and 5 ml filtered rumen at 37°C. Colonies were purified by selecting independent colonies directly from the plate and subculturing it.

### 2.3 Strain identification by MALDI‐TOF‐MS and 16S rRNA gene sequencing

Identification of bacterial colonies was done using matrix‐assisted laser desorption/ionization time‐of‐flight mass spectrometry (MALDI‐TOF‐MS) analysis as previously described (Seng et al., 2010). When MALDI‐TOF‐MS failed to identify the new organisms (score <1.7), 16S rRNA gene sequencing was performed using the fD1 and rP2 primers as formerly done (Drancourt, Berger, & Raoult, 2004). Each 16S rRNA sequence was compared with the nr database of the National Center for Biotechnology Information using the BLAST software (https://blast.ncbi.nlm.nih.gov). Compared to its phylogenetically closest species with standing in nomenclature, a 95% similarity threshold was used to define a new genus and a 98.65% similarity threshold was used to define a new species (Kim, Oh, Park, & Chun, 2014). The mass spectrum and 16S rRNA sequence of the newly isolated species were submitted in the URMITE (https://www.mediterranee-infection.com/article.php?laref=256&amp;titre=urms-database) and EMBL‐EBI databases, respectively.

### 2.4 Phylogenetic tree

For phylogenetic analysis, sequences of the phylogenetically closest species were obtained after performing a BLASTn search within the 16S rRNA database of “The All‐Species Living Tree" Project of Silva (The SILVA and “All‐species Living Tree Project (LTP)” taxonomic frameworks, 2013). Alignment was performed using CLUSTALW (Thompson, Higgins, & Gibson, 1994) and MEGA software (Kumar, Tamura, & Nei, 1994) was used for phylogenetic inferences generation using the maximum likelihood method.

### 2.5 Morphologic observation and growth conditions

Following Gram staining, bacterial cells were observed using a Leica DM 2500 photonic microscope (Leica Microsystems, Nanterre, France) with a 100X oil immersion lens. A wet mount was performed to determine motility of both bacteria and a Leica DM 1000 photonic microscope (Leica Microsystems) at a 1,000× total magnification. A Tecnai G20 (FEI company, Limeil‐Brevannes, France) electron microscope was used for bacterial cell imaging at an operating voltage of 60 kV, as previously described (Elsawi et al., 2017).

Culture of strains Marseille‐P2849^T^ and Marseille‐P3277^T^ was attempted using several growth conditions in order to determine the optimal ones. Culture assays were performed on 5% sheep blood‐enriched Columbia agar (bioMerieux) under different atmosphere including aerobic, anaerobic (GENbag Anaer, BioMerieux, France), and microaerophilic (GENbag Microaer, bioMerieux, Marcy‐l'Étoile, France) conditions. GENbag is commercially available, disposable sachet containing different chemical compounds (activated charcoal, sodium ascorbate, and others) used in the production of an anaerobic environment free of elemental oxygen gas (O_2_) or microaerophilic environment with 5% of elemental oxygen gas. Different growth temperatures (25, 28, 37, 45, 55°C), pH values (6–8.5), and NaCl concentrations (5–100 g/L) were also tested.

### 2.6 Biochemical analysis, fatty acid methyl ester analysis, and antibiotic susceptibility testing

Biochemical characteristics of the strains were investigated using API ZYM, 20A and 50CH strips (BioMérieux) according to the manufacturer's instructions. A 20‐min‐thermic shock of fresh colonies at 80°C was done in order to test sporulation. Catalase (BioMerieux) activity was determined in 3% hydrogen peroxide solution and oxidase activity was assessed using an oxidase reagent (Becton‐Dickinson).

Cellular fatty acid methyl ester (FAME) analysis was performed by gas chromatography/mass spectrometry (GC/MS). Two samples were prepared with approximately 17 mg of bacterial biomass per tube for strain Marseille‐P2849^T^ and 5 mg per tube for strain Marseille‐P3277^T^. Briefly, fatty acid methyl esters were separated using an Elite 5‐MS column and monitored by mass spectrometry (Clarus 500—SQ 8 S, Perkin Elmer, Courtaboeuf, France) as previously described (Dione et al., 2016). Spectral database search was performed using MS Search 2.0 operated with the Standard Reference Database 1A (NIST, Gaithersburg, USA) and the FAMEs mass spectral database (Wiley, Chichester, UK).

Antibiotic susceptibility was tested using the *E* test gradient strip method (BioMerieux) to determine the minimal inhibitory concentration (MIC) of each tested antibiotic on blood Colombia agar media (BioMerieux, France).

### 2.7 DNA extraction, genome sequencing, and assembly

Genomic DNA (gDNA) of strains Marseille‐P2849^T^ and Marseille‐P3277^T^ was extracted in two steps. A mechanical treatment was first performed using acid‐washed glass beads (G4649‐500g Sigma) and a FastPrep BIO 101 instrument (Qbiogene, Strasbourg, France) at maximum speed (6.5) for 90 s. Then after a 2 hr lysozyme incubation at 37°C, DNA was extracted on the EZ1 biorobot (Qiagen) with EZ1 DNA tissue kit according to the manufacturer's recommendations. Each gDNA was quantified by a Qubit assay with the high sensitivity kit (Life Technologies, Carlsbad, CA, USA) and was sequenced using the MiSeq technology (Illumina Inc, San Diego, CA, USA) with the Mate‐Pair strategy. Both gDNAs were barcoded in order to be mixed with 10 other projects with the Nextera Mate‐Pair sample prep kit (Illumina).

Each Mate‐Pair library was prepared with 1.5 µg of gDNA using the Nextera Mate‐Pair Illumina guide. Both gDNAs were simultaneously fragmented and tagged with a Mate‐Pair junction adapter. The fragmentation patterns were validated on an Agilent 2100 BioAnalyzer (Agilent Technologies Inc, Santa Clara, CA, USA) using a DNA 7500 labchip. DNA fragments size ranged between 1.5 and 11 kb. Strain Marseille‐P2849^T^ DNA fragments had an optimal size of 8.345 Kb, while strain Marseille‐P3277^T^ had an optimal size of 6.291 kb. No size selection was performed and 600 ng of tagmented fragments was circularized for strain Marseille‐P2849^T^ and 404.1 ng for strain Marseille‐P3277^T^. The circularized DNAs were mechanically sheared to small fragments with an optimal size at 960 bp on the Covaris device S2 in T6 tubes (Covaris, Woburn, MA, USA). The library profiles were visualized on a High Sensitivity Bioanalyzer LabChip (Agilent Technologies Inc, Santa Clara, CA, USA) and the final concentrations were measured at 12.3 and 3.9 nmol/L for strains Marseille‐P2849^T^ and Marseille‐P3277^T^, respectively.

The libraries were normalized at 2 nM and pooled. After a denaturation step and dilution at 15 pM, the pool of libraries was loaded onto the reagent cartridge and then onto the instrument along with the flow cell. Automated cluster generation and sequencing run were performed in a single 39‐hr run in a 2 × 151‐bp.

For strain Marseille‐P2849^T^, total information of 4.5 Gb was obtained from a 477 K/mm^2^ cluster density with a cluster passing quality control filters of 94.8% (8,444,000 passing filter paired reads). Within this run, the index representation for strain Marseille‐P2849^T^ was determined to be of 8.34%. For strain Marseille‐P3277^T^, total information of 6.3 Gb was obtained from a 673 K/mm^2^ cluster density with a cluster passing quality control filters of 95.4% (12,453,000 clusters). Within this run, the index representation for this strain was determined to be of 7.29%. The 769,472 and 907,611 paired reads of strains Marseille‐P2849^T^ and Marseille‐P3277^T^, respectively, were trimmed, assembled, annotated, and analyzed using the same pipeline adapted in our previous studies (Elsawi et al., 2017).

### 2.8 Genome annotation and analysis

Prodigal was used for open reading frame (ORF) prediction (Hyatt et al., 2010) with default parameters. We excluded predicted ORFs spanning a sequencing gap region (containing N). The bacterial proteome was predicted using BLASTP (*E*‐value of 1e03, coverage of 0.7 and identity percent of 30) against the clusters of orthologous groups (COGs) database. If no hit was found, we searched against the nr database (Clark, Karsch‐Mizrachi, Lipman, Ostell, & Sayers, 2016) using BLASTP with an *E*‐value of 1e03, coverage 0.7, and an identity percent of 30. An *E*‐value of 1e05 was used if the length of sequences was smaller than 80 amino acids. PFam conserved domains (PFAM‐A and PFAM‐B domains) were searched on each protein with the hhmscan tools analysis. RNAmmer (Lagesen et al., 2007) and tRNAScanSE tool (Lowe & Chan, 2016) were used to find ribosomal rRNAs genes and tRNA genes, respectively. ORFans were identified if all the BLASTP performed had negative results (*E*‐value inferior to 1e03 for ORFs with sequence size above 80 aa or *E*‐value inferior to 1e05 for ORFs with sequence length smaller than 80 aa). For data management and visualization of genomic features, Artemis (Carver, Harris, Berriman, Parkhill, & McQuillan, 2012) was used. We used the MAGI in‐house software to analyze the mean level of nucleotide sequence similarity at the genome level. It calculated the average genomic identity of gene sequences (AGIOS) among compared genomes (Ramasamy et al., 2014). This software combines the Proteinortho software (Lechner et al., 2011) for detecting orthologous proteins in pairwise genomic comparisons. Then, the corresponding genes were retrieved and the mean percentage of nucleotide sequence identity among orthologous ORFs was determined using the Needleman–Wunsch global alignment algorithm.

We also used the Genome‐to‐Genome Distance Calculator web service to calculate digital DNA:DNA hybridization estimates (dDDH) with confidence intervals under recommended settings (Formula 2, BLAST�) (Auch, Klenk, & Göker, 2010; Meier‐Kolthoff, Auch, Klenk, & Göker, 2013).

## 3 RESULTS

### 3.1 Strain identification by MALDI‐TOF‐MS and 16S rRNA sequencing

**Figure 1:**
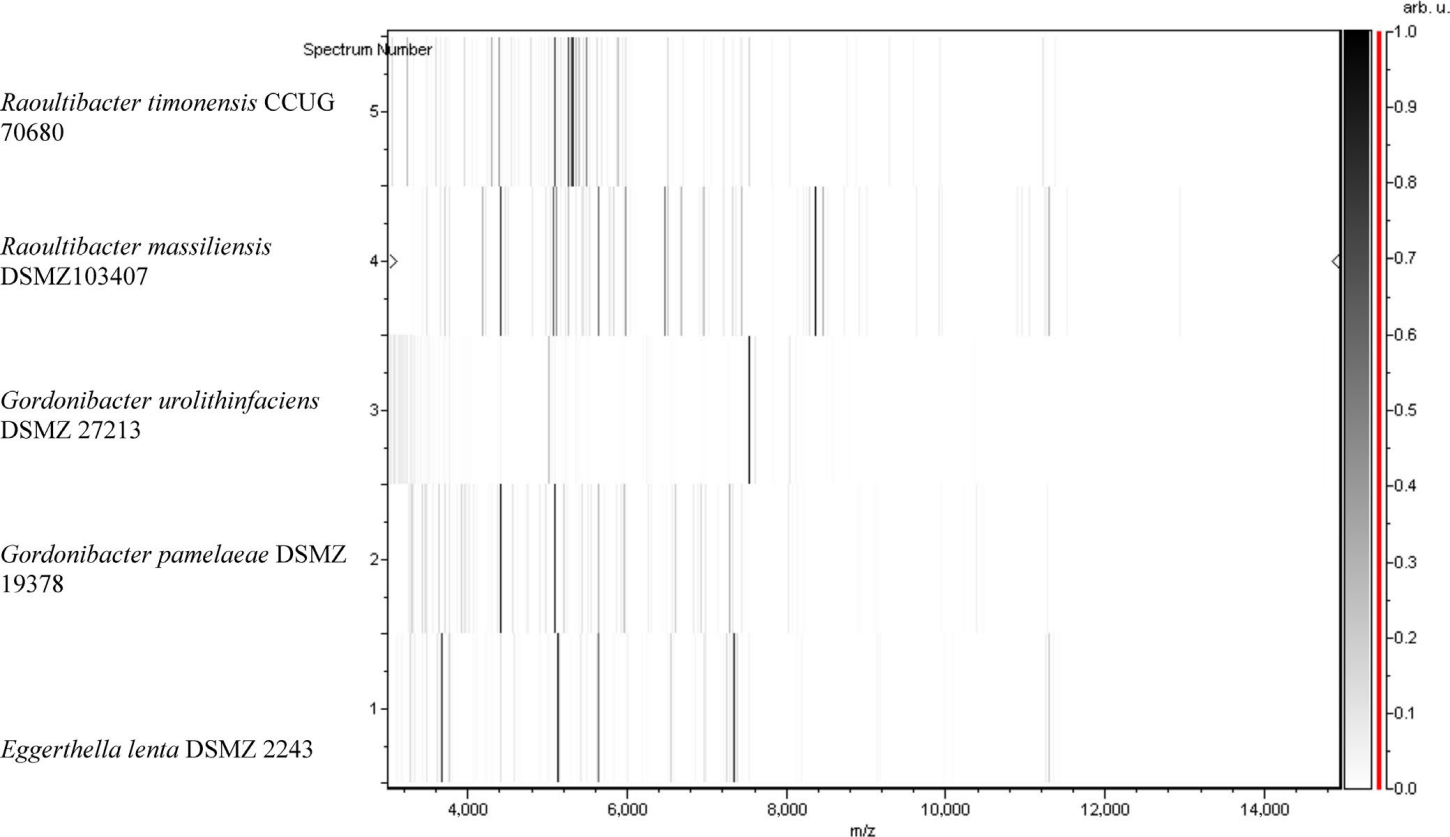
Gel view comparing *Raoultibacter massiliensis* gen. nov., sp. nov. strain Marseille‐P2849^T^ and strain *Raoultibacter timonensis* gen. nov., sp. nov. strain Marseille‐P3277^T^ with other closely related species present in our matrix‐assisted laser desorption/ionization time‐of‐flight mass spectrometry spectrum database. The gel view displays the raw spectra of loaded spectrum files arranged in a pseudo‐gel like look. The *x*‐axis records the *m*/*z* value. The left *y*‐axis displays the running spectrum number originating from subsequent spectra loading. The peak intensity is expressed by a gray scale scheme code. The color bar and the right *y*‐axis indicate the relation between the color of the peak and its intensity, in arbitrary units. Displayed species are indicated on the left

Matrix‐assisted laser desorption/ionization‐TOF‐MS failed to identify strains Marseille‐P2849^T^ and P3277^T^ at the genus and species levels (score <1.7). The spectra of strain Marseille‐P2849^T^ and Marseille‐P3277^T^ were added to our URMS database (Supporting Information Figure S1). A gel view comparing the available mass spectrum of the new isolated species to the mass spectrum of its phylogenetically close species was done (Figure 1). Mass spectrum of each organism was unique and did not match any other spectrum, confirming the novelty of both studied strains.

**Figure 2:**
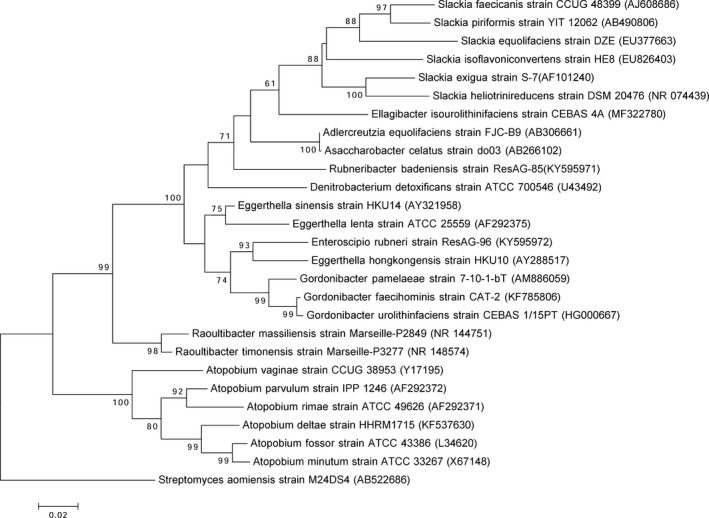
Phylogenetic tree highlighting the position of *Raoultibacter massiliensis* strain gen. nov., sp. nov. strain Marseille‐P2849^T^ and *Raoultibacter timonensis* gen. nov., sp. nov. strain Marseille‐P3277^T^ relative to other closely related species. Strains and their GenBank accession numbers of 16S rRNA gene are indicated in brackets. Sequences were aligned using ClustalW, with default parameters and phylogenetic inferences obtained using the neighbor‐joining method with 500 bootstrap replicates, within MEGA7 software. The scale bar represents a 2% nucleotide sequence divergence

Strain Marseille‐P2849^T^ exhibited a 91.4% 16S rRNA gene sequence similarity with *Gordonibacter urolithinfaciens* strain CEBAS 1/15 PT (GenBank accession number HG000667), the phylogenetically closest species with standing in nomenclature (Figure 2), suggesting it as a new genus within the family *Eggerthellaceae*, namely *Raoultibacter*. As for strain Marseille‐P3277^T^, it exhibited a 97.96% sequence similarity with strain Marseille‐P2849^T^, suggesting it as a new species within the *Raoultibacter* genus. The 16S rRNA sequences of strains Marseille‐P2849^T^ and Marseille‐P3277^T^ were deposited in EMBL‐EBI under accession numbers LT576395 and LT623894, respectively.

### 3.2 Phenotypic characteristics and biochemical features

**Figure 3:**
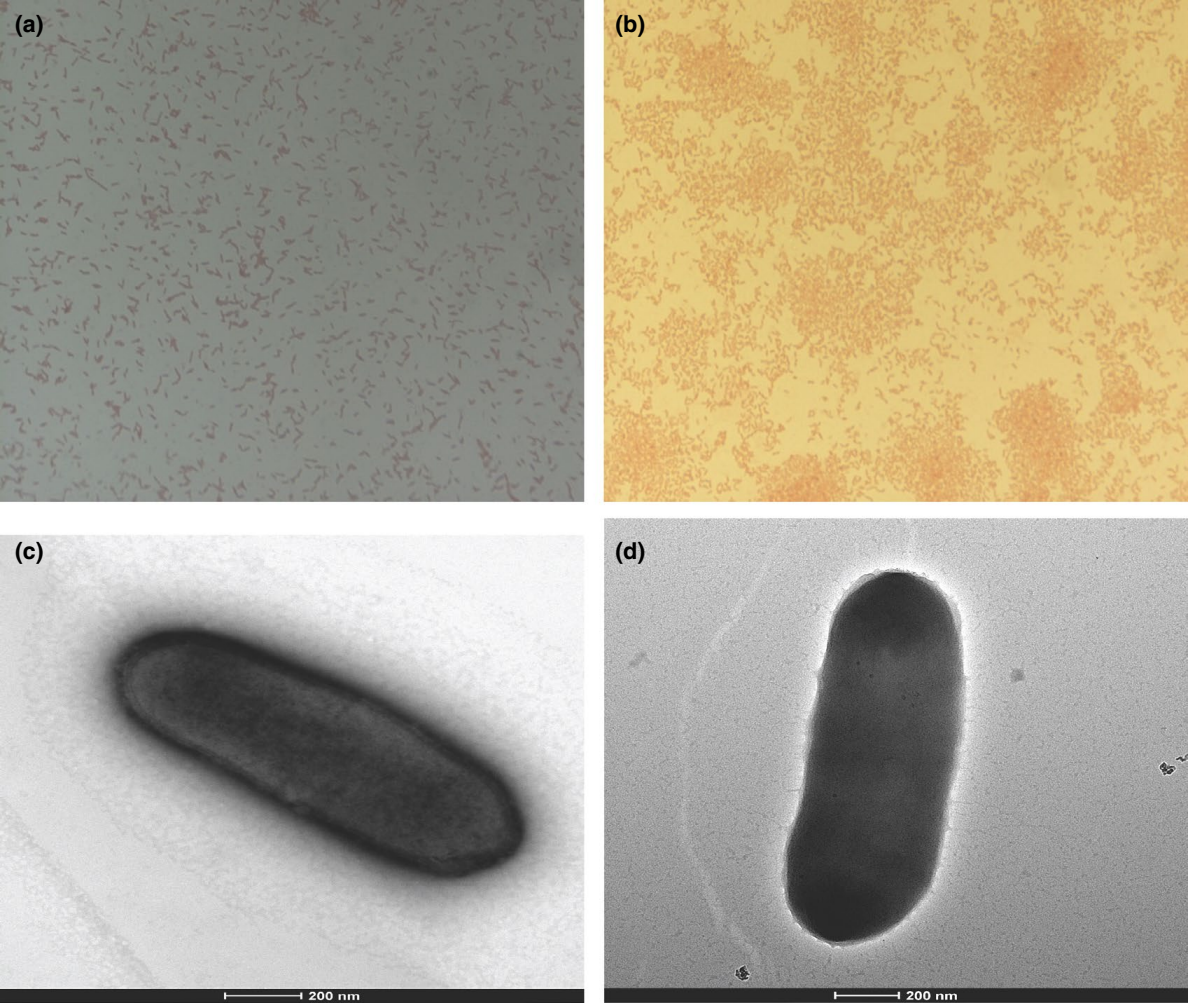
Gram staining of (a) *Raoultibacter massiliensis* gen. nov., sp. nov. strain Marseille‐P2849^T^ and (b) *Raoultibacter timonensis* gen. nov., sp. nov strain Marseille‐P3277^T^. Transmission electron microscopy images of *R. massiliensis* gen. nov., sp. nov. strain Marseille‐P2849^T^ (c) and *R. timonensis* gen. nov., sp. nov strain Marseille‐P3277^T^ (d) using a Tecnai G20 transmission electron microscope (FEI Company). The scale bar represents 200 nm

Strains Marseille‐P2849^T^ and Marseille‐P3277^T^ form translucent microcolonies on 5% sheep blood‐enriched Columbia agar (bioMérieux) with a mean diameter ranging from 0.1 to 0.4 mm. The growth of both strains was observed in anaerobic and microaerophilic atmospheres at 28, 37, and 45°C but optimally under anaerobic conditions at 37°C after 48 hr of incubation. No growth was obtained at 55°C or in aerobic atmosphere. Bacterial cells were motile, Gram‐positive (Figure 3a,b), and nonsporeforming rod. Strain Marseille‐P2849^T^ cells had a length ranging between 0.8 and 1.2 μm with a mean diameter ranging from 0.4 to 0.6 μm (Figure 3c,d). As for strain Marseille‐P3277^T^, its cells were 1–2 μm long with a mean diameter ranging from 0.35 to 0.44 μm. Both strains were catalase positive, oxidase negative, tolerated pH levels ranging between 6 and 8.5 and could not sustain NaCl concentration >5 g/L. The classification and general features of strains Marseille‐P2849^T^ and Marseille‐P3277^T^ are summarized in Table 1.

**Table 1:** Classification and general features of *Raoultibacter massiliensis* strain Marseille‐P2849^T^ and *Raoultibacter timonensis* strain Marseille‐P3277^T^

Properties	Term	
Current classification	Domain: *Bacteria*	Domain: *Bacteria*
	Phylum: *Actinobacteria*	Phylum: *Actinobacteria*
	Class: *Coriobacteriia*	Class: *Coriobacteriia*
	Order: *Eggerthellales*	Order: *Eggerthellales*
	Family: *Eggerthellaceae*	Family: *Eggerthellaceae*
	Genus: *Raoultibacter*	Genus: *Raoultibacter*
	Species: *R. massiliensis*	Species: *R. timonensis*
	Type strain: Marseille‐P2849^T^	Type strain: Marseille‐P3277^T^
Gram‐stain	Positive	Positive
Cell shape	Rod	Rod
Motility	Motile	Motile
Sporulation	Nonsporulating	Nonsporulating
Temperature range	25–45°C	25–4°C
Optimum temperature	37°C	37°C
Oxygen requirement	Anaerobic or microaerophilic	Anaerobic or microaerophilic
Biotic relationship	Free living	Free living
Isolation	Human feces	Human feces

Using an API® 50CH strip (bioMérieux), positive reactions were observed for both strains for glycerol, d‐ribose, d‐galactose, d‐glucose, d‐fructose, d‐mannose, d‐mannitol, d‐arabitol, *N*‐acetylglucosamine, amygdaline, arbutin, esculin ferric citrate, salicin, d‐maltose, d‐lactose, d‐saccharose, d‐trehalose, d‐melezitose, gentiobiose, d‐tagatose, and potassium gluconate. In addition, positive reactions were observed for strain Marseille‐P2849^T^ with amidon and potassium 5‐ketogluconate, and for strain Marseille‐P3277^T^ with methyl‐αd‐glucosamine, d‐cellobiose, and d‐turanose (Table 2). Negative reactions were observed for both strains for erythritol, A‐arabinose, l‐Arabinose, d‐Xylose, l‐xylose, d‐adonitol, methyl‐βd‐xylopyranoside, l‐sorbose, l‐rhamnose, dulcitol, inositol, methyl‐αd‐mannopyranoside, methyl‐αd‐glucopyranoside, d‐cellobiose, d‐melibiose, inulin, d‐raffinose, glycogen, xylitol, d‐turanose, d‐xylose, d‐fucose, l‐fucose, l‐arabitol, and potassium 2‐ketogluconate.

**Table 2:** Differential characteristics of *Raoultibacter massiliensis* strain Marseille‐P2849^T^ (1), *Raoultibacter timonensis* strain Marseille‐P3277^T^ (2), *Gordonibacter pamelaeae* strain 7‐10‐1‐b^T^ (Würdemann et al., 2009) (3), *Gordonibacter urolithinfaciens* CEBAS 1/15P^T^ (Selma, Tomás‐Barberán, Beltrán, García‐Villalba, & Espín, 2014) (4), *Eggerthella sinensis* HKU14^T^ (Lau et al., 2004) (5), *Paraeggerthella hongkongensis* strain HKU10^T^ (Lau et al., 2004; Würdemann et al., 2009) (6), *Eggerthella lenta* JCM 997^T^ (Wade et al., 1999) (7), *Adlercreutzia equolifaciens* strain DSM19450^T^ (Maruo, Sakamoto, Ito, Toda, & Benno, 2008) (8), *Asaccharobacter celatus* strain do03^T^ (Minamida et al., 2008) (9), *Cryptobacterium curtum* strain 12‐3^T^ (Nakazawa et al., 1999) (10), *Denitrobacterium detoxificans* strain NPOH1^T^ (Anderson, Rasmussen, Jensen, & Allison, 2000) (11), *Enterorhabdus mucosicola* strain Mt1B8^T^ (Clavel et al., 2009) (12), *Slackia exigua* strain S‐7^T^ (Wade et al., 1999) (13), *Ellagibacter isourolithinifaciens* CEBAS 4A^T^ (Beltrán, Romo‐Vaquero, Espín, Tomás‐Barberán, & Selma, 2018) (14), *Rubneribacter badeniensis* ResAG‐85^T^ (Danylec et al., 2018) (15)

	1	2	3	4	5	6	7	8	9	10	11	12	13	14	15
Cell length (µm)	0.8–1.2/0.4–0.6	0.8–1.2	1.2/0.5	1.57/0.61	NA	NA	0.2–0.4/0.2–2.0	0.6–0.76/1.5–2.7	0.45/2.3–2.7	0.4/0.8–1	0.5–1.0/1.0–1.5	0.5/2.0	0.5/1.0	0.5/1.5	0.3/1
Oxygen requirement	Anaerobe and micro aerophilic	Anaerobe and micro aerophilic	Strict anaerobe	Strict anaerobe	Strict anaerobe	Strict anaerobe	Strict anaerobe	Strict anaerobe	Strict anaerobe	Strict anaerobe	Strict anaerobe	Strict anaerobe	Strict anaerobe	Strict anaerobe	Strict anaerobe
Gram‐stain	positive	Positive	Positive	Positive	Positive	Positive	Positive	Positive	Positive	Positive	Positive	Positive	Positive	Positive	Positive
Indole	+	+	Na	Na	−	−	−	Na	Na	−	−	−	−	−	−
Motility	+	+	+	+	−	−	−	−	Na	−	−	Na	−	−	−
Endospore formation	−	−	−	−	−	−	−	−	−	−	−	−	−	−	Na
Production of															
Nitrate reductase	−	Na	−	−	−	−	+	−	−	−	+		−	−	−
Catalase	+	+	+	+	+	+	V	Na	−	−	Na	−	−	−	−
Urease	−	−	−	Na	−	−	−	−	Na	−	Na	Na	−	−	−
Phosphatase alkaline	−	−	−	−	−	−	−	Na	−	Na	Na	Na	Na	Na	Na
Acid from															
l‐fucose	−	Na	−	+	−	−	−	Na	−	−	Na	−	−	Na	Na
d‐ribose	+	+	Na	Na	−	Na	+	Na	−	−	Na	−	−	Na	Na
l‐arabinose	−	−	Na	−	−	−	+	Na	−	−	Na	Na	−	−	−
d‐mannitol	+	+	Na	Na	Na	Na	Na	Na	−	−	Na	Na	−	Na	−
d‐mannose	+	+	−	−	−	−	−	Na	−	−	Na	Na	−	−	−
Raffinose	+	+	−	−	−	−	−	Na	−	Na	Na	Na	−	Na	−
l‐rhamnose	−	+	−	−	−	+	+	Na	−	−	Na	Na	−	Na	−
Trehalose	+	+	−	−	−	−	−	Na	−	−	Na	Na	−	Na	−
d‐glucose	+	+	+	−	−	−	+	−	−	−	Na	Na	−	Na	−
d‐fructose	+	+	Na	+	Na	Na	Na	Na	−	−	Na	Na	−	Na	Na
d‐maltose	+	+	Na	Na	Na	Na	Na	Na	−	−	Na	Na	−	Na	−
d‐lactose	+	+	Na	Na	Na	Na	Na	Na	−	−	Na	Na	−	Na	−
DNA G+C content (mol%)	59.01	59.6	66.4	66.4	64.9	61.1	63.8	63.5	63	50.9	59.5	64.2	62.1	59.6	65.1
Isolation source	Human feces	Human feces	Human colon	Human feces	Blood culture	Blood culture	Human feces	Human feces	Rat cecum	Human oral cavities	Bovine rumen	Ileal mucosa of mice	Human oral lesions	Human feces	Human feces

Note

NA: data not available; v: variable.

Using an API® 20A strip (bioMérieux), both strains produced indole. In addition, positive reactions were observed for d‐glucose, d‐mannitol, d‐lactose, d‐saccharose, d‐maltose, salicin, l‐arabinose, gelatine, d‐mannose, esculin ferric citrate, d‐cellobiose d‐melezitose, d‐raffinose, d‐sorbitol, and d‐trehalose for both strains. Positive reaction was observed for strain Marseille‐P3277^T^, but not Marseille‐P2849^T^, with l‐rhamnose. No reaction was obtained for urease and d‐xylose for both strains.

Using an API® ZYM strip (bioMérieux), both strains exhibited esterase (C4), esterase lipase (C8), lipase (C14), leucine arylamidase, valine arylamidase, cystine arylamidase, phosphatase acid, and naphthol phosphohydrolase activities but no phosphatase alkaline was observed. In addition, positive reactions were observed for strain Marseille‐P3277^T^ with trypsin, α‐chymotrypsin, α‐galactosidase, β‐galactosidase, β‐glucuronidase, α‐glucosidase, β‐glucosidase, *N*‐acetyl‐β‐glucosaminidase, and α‐mannosidase. An α‐fucosidase activity was observed only for strain Marseille‐P2849^T^.

The major fatty acids identified for strains Marseille‐P2849^T^ and Marseille‐P3277^T^ were 9‐octadecenoic acid (Cl8:ln9, 36% and 38%, respectively), hexadecanoic acid (C16:0, 18% and 25%), and tetradecanoic acid (Cl4:0, 13% and 11%; Table 3). Strain Marseille‐P3277^T^ exhibited unusually long chain fatty acids (C20:4n6 and C20:5n3).

**Table 3:** Cellular fatty acid composition (%) of strain Marseille‐P2849^T^ and strain Marseille‐P3277^T^ compared with other type strains of closely related species: 1, strain Marseille‐P2849^T^; 2, strain Marseille‐P3277^T^; 3, *Gordonibacter urolithinfaciens* strain CEBAS 1/15P^T^; 4, *Gordonibacter pamelaeae* strain 7‐10‐1‐b^T^; 5, *Paraeggerthella hongkongensis* DSM 16106^T^; 6, *Eggerthella lenta* DSM 2243^T^; 7, *Eggerthella sinensis* DSM 16107^T^

Fatty acids		1	2	3	4	5	6	7
C18:1n9	9‐octadecenoic acid	36.4	38.1	27	6.8	55.1	42.3	36.6
C16:0	Hexadecanoic acid	18.2	25.4	4.4	4.5	7.1	6.7	7.6
C14:0	Tetradecanoic acid	12.7	10.9	5.2	16.3	6.9	12.5	7.7
C15:0 anteiso	12‐methyl‐tetradecanoic acid	7.3	1.4	22.7	36.9	1.1	16.3	21.2
C18:2n6	9,12‐octadecadienoic acid	6.7	9	ND	ND	1.4	ND	ND
C18:0	Octadecanoic acid	3.4	5.7	5.6	1.5	4.7	1.4	1.5
C18:1n7	11‐octadecenoic acid	3.2	3.7	1.4	ND	4.3	2.6	2.3
C15:0 iso	13‐methyl‐tetradecanoic acid	2.8	2.8	3.6	5.5	0	1.1	0
C12:0	Dodecanoic acid	1.8	1.8	TR	5	7.7	2.9	1.1
C13:0 iso	11‐methyl‐dodecanoic acid	1.5	ND	TR	2	ND	ND	ND
C14:0 iso	12‐methyl‐tridecanoic acid	1.4	ND	13.4	18.3	0	7.5	17.1
C15:0	Pentadecanoic acid	1.2	1.1	ND	ND	ND	ND	ND
13:0 anteiso	10‐methyl‐dodecanoic acid	1.1	ND	ND	ND	ND	ND	1
C20:4n6	5,8,11,14‐eicosatetraenoic acid	TR	1.2	ND	ND	ND	ND	ND
C20:5n3	5,8,11,14,17‐eicosapentaenoic acid	ND	TR	ND	ND	ND	ND	ND
C5:0 iso	3‐methyl‐butanoic acid	TR	ND	ND	ND	ND	ND	ND
C13:0	Tridecanoic acid	TR	ND	ND	ND	ND	ND	ND
C16:1n7	9‐hexadecenoic acid	TR	ND	2	3.2	8.8	4.4	2.6

Note

Values represent the percentage of total identified fatty acid methyl esters only (aldehydes, dimethyl acetals and unidentified “summed features” described previously were not included). Data of the close species were taken as reported by Selma et al. (2014).

ND: not detected; TR: trace amounts <1%.

Among tested antibiotics, strains Marseille‐P2849^T^ and Marseille‐P3277^T^ were susceptible to amoxicillin (MIC 0.50 and 1 µg/ml, respectively), imipenem (0.047 and 0.047 µg/ml), metronidazole (0.023 and 0.064 µg/ml), rifampicin (0.003 and 0.008 µg/ml), and erythromycin (0.32 and 0.016 µg/ml). Both strains were resistant to daptomycin, minocycline, amikacin, vancomycin, and cefotaxime.

### 3.3 Genomic properties

**Figure 4:**
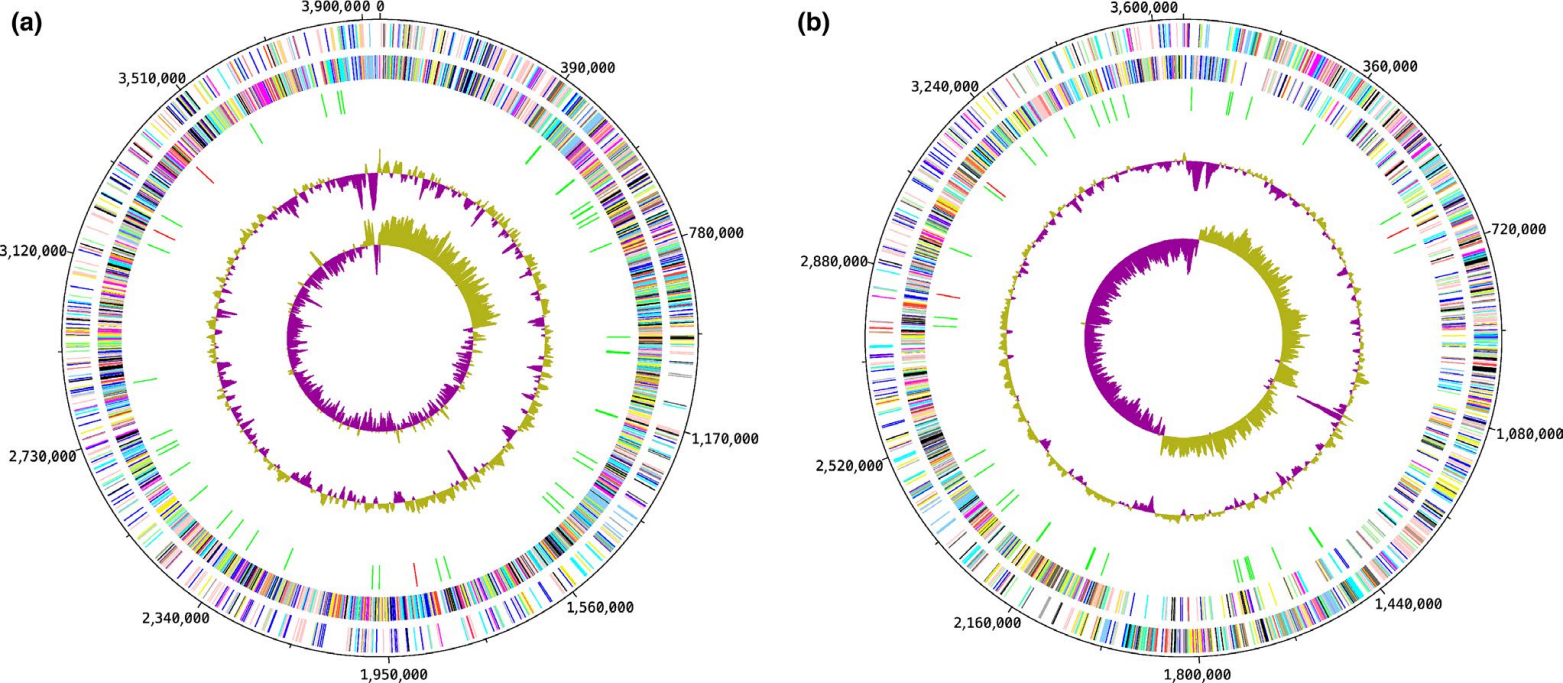
Graphical circular map of the genome of (a) *Raoultibacter massiliensis* gen. nov., sp. nov. strain Marseille‐P2849^T^ and (b) strain *Raoultibacter timonensis* gen. nov., sp. nov. strain Marseille‐P3277^T^. From the outside to the center, contigs (red/gray), clusters of orthologous groups (COGs) category of genes on the forward strand (three circles), genes on the forward strand (blue circle), genes on the reverse strand (red circle), COG category of genes on the reverse strand (three circles), G+C skew (purple indicates positive values and olive negative values)

The draft genome of strain Marseille‐P2849^T^ was 3,657,161‐bp long with a G+C content of 59.02 mol% (Table 4; Figure 4a). It was composed of nine scaffolds (35 contigs). Of the 3,073 predicted genes, 3,025 were protein‐coding genes and 48 were RNAs (one complete rRNA operon and 45 tRNA genes). A total of 2,365 proteins (76.86%) were assigned to COGs and 253 genes were identified as ORFans (8.23%). Six genes were associated with polyketide synthases (PKS) or nonribosomal peptide synthetases (NRPS; 0.18%) and 470 genes were associated with virulence (15.29%). As for strain Marseille‐P3277^T^, the genome size was 4,000,215‐bp long with a 59.9 mol% G+C content (Figure 4b). It was composed of 21 scaffolds (composed of 84 contigs). Of the 3,284 predicted genes, 3,232 were protein‐coding genes and 52 were RNAs (one complete rRNA operon and 49 tRNA genes). A total of 2,562 proteins (78.01%) were assigned to COGs and 323 genes were identified as ORFans (9.83%). The genome of strain Marseille‐P3277^T^ contained 14 genes associated with PKS or NRPS (0.45%) and 481 genes associated with virulence (14.64%). The genome statistics are presented in Table 4, and the distribution of genes into COGs functional categories is summarized in Table 5.

**Table 4:** Nucleotide content and gene count levels of the genome of strain *Raoultibacter massiliensis* Marseille‐P2849^T^ and *Raoultibacter timonensis* strain Marseille‐P3277^T^

	*Raoultibacter massiliensis*		*Raoultibacter timonensis*	
	Number	Percent (%)	Number	Percent (%)
Size (bp)	3,657,161	100	4,000,215	100
Number of G+C	2,158,456	59	2,396,128	59.9
Number total of genes	3,073	100	3,284	100
Total number of protein‐coding genes	3,025	98.4	3,232	99.33
Total number of RNA Genes	48	1.56	52	1.58
Total number of tRNA Genes	45	1.6	48	1.46
Total number of rRNA (5S, 16S, 23S) Genes	3	0.1	3	0.12
Coding sequence gene protein size	3,156,910	86.3	3,498,188	87.45
Number of proteins associated with clusters of orthologous groups	2,365	77	2,562	78.01
Number of proteins associated with orfan	253	8,23	323	9.83
Number of proteins with peptide signal	385	12,5	512	15.59
Number of genes associated with PKS or NRPS	6	0.18	14	0.45
Number of genes associated with virulence	470	15.3	481	14.64
Number of proteins with TMH	855	27.8	940	28.62

Notes

The total is based on either the size of the genome in base pairs or the total number of protein‐coding genes in the annotated genome.

**Table 5:** Number of genes associated with the 25 general clusters of orthologous group (COG) functional categories

Code	*Raoultibacter massiliensis*		*Raoultibacter timonensis*		Description
	Value	% of total	Value	% of total	
[J]	134	4.43	142	4.39	Translation
[A]	0	0	0	0	RNA processing and modification
[K]	264	8.73	291	9.01	Transcription
[L]	102	3.37	95	2.94	Replication, recombination and repair
[B]	0	0	0	0	Chromatin structure and dynamics
[D]	23	0.76	16	0.5	Cell cycle control. mitosis and meiosis
[Y]	0	0	0	0	Nuclear structure
[V]	64	2.12	57	1.76	Defense mechanisms
[T]	181	5.98	214	6.62	Signal transduction mechanisms
[M]	121	4	115	3.56	Cell wall/membrane biogenesis
[*N*]	8	0.26	9	0.28	Cell motility
[Z]	0	0	0	0	Cytoskeleton
[W]	0	0	0	0	Extracellular structures
[U]	18	0.6	20	0.62	Intracellular trafficking and secretion
[O]	83	2.74	86	2.66	Posttranslational modification, protein turnover, chaperones
[X]	5	0.17	2	0.06	Mobilome: prophages, transposons
[C]	409	13.52	477	14.76	Energy production and conversion
[G]	118	3.9	132	4.08	Carbohydrate transport and metabolism
[E]	160	5.29	171	5.29	Amino acid transport and metabolism
[F]	55	1.82	58	1.79	Nucleotide transport and metabolism
[H]	65	2.15	69	2.13	Coenzyme transport and metabolism
[I]	49	1.61	55	1.7	Lipid transport and metabolism
[P]	120	3.97	139	4.3	Inorganic ion transport and metabolism
[Q]	18	0.6	21	0.65	Secondary metabolites biosynthesis, transport and catabolism
[R]	214	7.07	226	6.99	General function prediction only
[S]	154	5.09	167	5.18	Function unknown
–	660	21.82	670	20.73	Not in COGs

Note

The total is based on either the size of the genome in base pairs or the total number of protein‐coding genes in the annotated genome.

### 3.4 Genomic comparison

**Figure 5:**
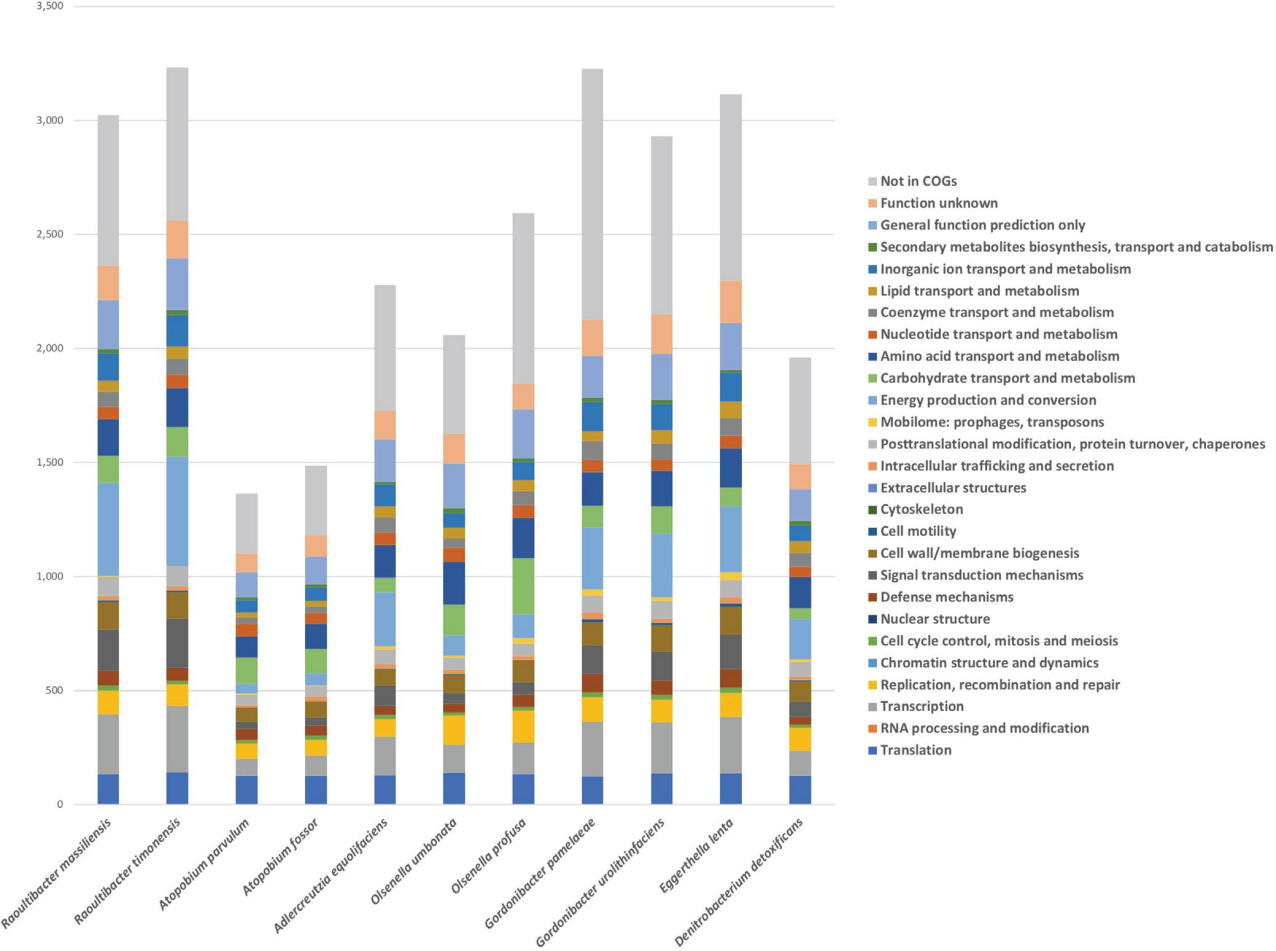
Distribution of functional classes of predicted genes according to the clusters of orthologous groups of proteins of *Raoultibacter massiliensis* gen. nov., sp. nov. strain Marseille‐P2849^T^ and strain *Raoultibacter timonensis* gen. nov., sp. nov. strain Marseille‐P3277^T^ among other closely related species

The draft genome sequence structure of strains Marseille‐P2849^T^ and Marseille‐P3277^T^ is summarized in Figure 4. The draft genome sequence of strain Marseille‐P2849^T^ was larger than that of *G. urolithinfaciens*,* Atopobium fossor*,* Denitrobacterium detoxificans*,* Atopobium parvulum*,* Olsenella profusa*,* Olsenella uli*,* E. lenta*, and *Gordonibacter pamelaeae* (3.29, 1.66, 2.45, 1.54, 2.72, 2.05, 3.63, and 3.61 Mb, respectively) but smaller than that of strain Marseille‐P3277^T^ (3.94 Mb, Table 6). The G+C content of strains Marseille‐P2849^T^ and Marseille‐P3277^T^ was larger than those of *A*. *fossor* and *A. parvulum* (59.02 and 59.9 vs. 45.4 and 45.7, respectively), but smaller than those of *G. urolithinfaciens*,* D. detoxificans*,* G. pamelaeae*,* E. lenta*,* O. profusa*, and *O. uli* (66.1 59.5%, 64.0%, 64.2%, 64.2%, and 64.7%, respectively). The gene content of strain Marseille‐P2849^T^ was smaller than that of strain Marseille‐P3277^T^ (3,073 and 3,284, respectively), but larger than that of *G. urolithinfaciens*,* A. fossor*,* G. pamelaeae*,* D. detoxificans*,* A. parvulum*,* O. profusa*, and *E. lenta* (2,836, 1,487, 2,027, 1,762, 1,353, 2,650, and 3,070, respectively). The distribution of functional classes of predicted genes of strains Marseille‐P2849^T^ and Marseille‐P3277^T^ according to the COGs of proteins is summarized in Figure 5.

**Table 6:** Genome comparison of species closely related to *Raoultibacter massiliensis* strain Marseille‐P2849^T^ and *Raoultibacter timonensis* strain Marseille‐P3277^T^

Species	INSDC identifier	Size (Mb)	G+C (mol %)	Gene Content
*Raoultibacter massiliensis* strain Marseille‐P2849^T^	FZQX00000000	3.65	59.01	3,021
*Raoultibacter timonensis* strain Marseille‐P3277^T^	OEPT00000000	3.94	59.6	3,277
*Eggerthella lenta strain DSM 2243* ^T^	NC_013204.1	3.63	64.2	3,146
*Denitrobacterium detoxificans* strain NPOH1^T^	NZ_CP011402.1	2.45	59.5	2,023
*Gordonibacter pamelaeae* strain 7‐10‐1‐b^T^	NC_021021.1	3.61	64	3,352
*Atopobium fossor* strain ATCC 43386^T^	AXXR00000000.1	1.66	45.4	1,505
*Atopobium parvulum* strain DSM 20469^T^	NC_013203.1	1.54	45.7	1,406
*Olsenella profusa* strain DSM 13989^T^	AWEZ00000000.1	2.72	64.2	2,707
*Olsenella uli* strain ATCC 49627^T^	CP002106.1	2.05	64.7	1,822
*Adlercreutzia equolifaciens* strain DSM19450^T^	NC_022567.1	2.86	63.5	2,326
*Gordonibacter urolithinfaciens* strain CEBAS 1/15P^T^	NZ_LT900217.1	3.29	66.1	2,836

Note

INSDC: International Nucleotide Sequence Database Collaboration.

Strain Marseille‐P2849^T^ shared 1,542, 1,370, 555, 571, 1,069, 693, 683, 1,084, 1,404, and 911 orthologous proteins with strain Marseille‐P3277^T^, *G. urolithinfaciens*,* A. parvulum*,* A. fossor*,* Adlercreutzia equolifaciens*,* Olsenella umbonata*,* O. profusa*,* G. pamelaeae*,* E. lenta*, and *D. detoxificans*, respectively. The AGIOS values among the eight most closely related species ranged between 58.12% and 81.35%. When compared to these eight species, strain Marseille‐P2849^T^ AGIOS values ranged from 58.97% with *A. fossor* to 73.75% with *G. pamelaeae*. Similarly, strain Marseille‐P3277^T^ exhibited AGIOS values ranging from 58.95% with *A. fossor* to 74.19% with *G. pamelaeae* (Table 7). The AGIOS values obtained for strains Marseille‐P2849^T^ and Marseille‐P3277^T^, between 58.12% and 81.35%, support their new species status.

**Table 7:** Number of orthologous proteins shared between genomes (upper right) and AGIOS values (%) obtained (lower left)

	*Raoultibacter massiliensis*	*Raoultibacter timonensis*	*Atopobium parvulum*	*Atopobium fossor*	*Adlercreutzia equolifaciens*	*Olsenella umbonata*	*Olsenella profusa*	*Gordonibacter pamelaeae*	*Gordonibacter urolithinfaciens*	*Eggerthella lenta*	*Denitrobacterium detoxificans*
*Raoultibacter massiliensis*	3,025	1,542	555	571	1,069	693	683	1,084	1,370	1,404	911
*Raoultibacter timonensis*	81.25	3,232	529	552	1,029	647	643	1,086	1,057	1,373	863
*Atopobium parvulum*	59.35	59.27	1,363	706	523	772	769	412	434	576	534
*Atopobium fossor*	58.97	58.95	66.76	1,487	546	774	754	425	500	605	541
*Adlercreutzia equolifaciens*	69.69	70.09	58.3	58.12	2,278	649	621	770	609	1,094	861
*Olsenella umbonata*	64.29	64.82	63.57	62.66	66.2	2,059	909	496	409	719	645
*Olsenella profusa*	63.81	64.37	62.95	62.73	65.97	74.21	2,593	501	483	704	628
*Gordonibacter pamelaeae*	73.75	74.19	58.95	58.73	74.46	67.76	66.84	3,228	1,426	1,056	644
*Gordonibacter urolithinfaciens*	72.85	73.58	55.47	56.14	74.04	66.7	66.1	91.6	2,793	987	745
*Eggerthella lenta*	72.92	73.35	58.39	58.06	73.45	67	66.14	81.35	80.48	3,116	921
*Denitrobacterium detoxificans*	68.46	68.75	60.29	60.14	68.84	64.956	64.84	70.75	71.05	69.92	1,960

Note

The number of proteins per genome is indicated in bold. The strains of the species included in the genomic analysis were given in Table 6.

In addition, dDDH values obtained between strain Marseille‐P2849^T^, strain Marseille‐P3277^T^, *G. urolithinfaciens*,* A. parvulum*,* A. fossor*,* A. equolifaciens*,* O. umbonata*,* O. profusa*,* G. pamelaeae*,* E. lenta*, and *D. detoxificans* were of 25.2% (22.9–27.7), 22.4% (20.2–24.9), 28.1% (25.8–30.6), 30.7% (28.3–33.2), 20.3% (18.1–22.8), 20.8% (18.6–23.3), 18.6% (16.5–21), 24.5% (22.2–27), 23.6% (21.3–26.1), and 19.1% (16.9–21.5), respectively (Table 8). These dDDH values were lower than the 70%, value threshold for species demarcation, thus confirming that the two studied strains are representative of two new species (Meier‐Kolthoff et al., 2013).

**Table 8:** Digital DNA–DNA hybridization values (%) obtained by comparison of *Raoultibacter massiliensis* strain Marseille‐P2849^T^ and *Raoultibacter timonensis* strain Marseille‐P3277^T^ with other closely related species using the GGDC formula 2 software (DDH estimates based on identities/HSP length)[Link], upper right

	*Raoultibacter massiliensis*	*Raoultibacter timonensis* (%)	*Atopobium parvulum* (%)	*Atopobium fossor* (%)	*Adlercreutzia equolifaciens* (%)	*O. umbonata* (%)	*Olsenella profusa* (%)	*Gordonibacter pamelaeae* (%)	*Gordonibacter urolithinfaciens* (%)	*Eggerthella lenta* (%)	*Denitrobacterium detoxificans* (%)
*Raoultibacter massiliensis*	100	25.2 (22.9–27.7)	28.1 (25.8–30.6)	30.7 (28.3–33.2)	20.3 (18.1–22.8)	20.8 (18.6–23.3)	18.6 (16.5–21)	24.5 (22.2–27)	22.4 (20.2–24.9)	23.6 (21.3–26.1)	19.1 (16.9–21.5)
*Raoultibacter timonensis*		100	28 (25.7–30.5)	30.1 (27.7–32.6)	20.4 (18.2–22.9)	21.5 (19.2–23.9)	19 (16.8–21.4)	22.9 (20.6–25.3)	22.3 (20–24.8)	22 (19.7–24.4)	19.1 (17–21.5)
*Atopobium parvulum*			100	20.3 (18.1–22.8)	22.6 (20.3–25)	26.2 (23.9–28.7)	24 (21.7–26.5)	25.3 (23–27.8)	25.7 (23.4–28.2)	25.8 (23.5–28.3)	24.4 (22.1–26.9)
*Atopobium fossor*				100	23.7 (21.4–26.2)	21.3 (19–23.7)	19.8 (17.6–22.2)	26.8 (24.5–29.3)	27.1 (24.8–29.6)	26.4 (24–28.9)	25.2 (22.9–27.7)
*Adlercreutzia equolifaciens*					100	18.2 (16.1–20.6)	17.9 (15.8–20.3)	22.4 (20.1–24.8)	21.5 (19.2–23.9)	21.5 (19.3–24)	19.5 (17.4–21.9)
*Olsenella umbonata*						100	21.7 (19.5–24.2)	18.2 (16.1–20.6)	19.2 (17–21.6)	20.4 (18.1–22.8)	33.7 (31.3–36.2)
*Olsenella profusa*							100	18 (15.9–20.4)	18.6 (16.4–21)	19.3 (17.1–21.7)	22.3 (20–24.8)
*Gordonibacter pamelaeae*								100	53 (50.3–55.7)	29.4 (27–31.9)	19.7 (17.5–22.1)
*Gordonibacter urolithinfaciens*									100	25.9 (23.5–28.4)	19.8 (17.6–22.2)
*Eggerthella lenta*										100	20.2 (17.9–22.6)
*Denitrobacterium detoxificans*											100

The confidence intervals indicate the inherent uncertainty in estimating DNA hybridization estimates (DDH) values from intergenomic distances based on models derived from empirical test data sets (which are always limited in size).

## 4 DISCUSSION

Culturomics is a high‐throughput culture approach that enabled the isolation of approximately 1,057 bacterial species including 247 new species from the human gut in our laboratory (Jean‐Christophe Lagier et al., 2016). Along with the development of culturomics, a new polyphasic approach, taxonogenomics, was developed in order to describe novel bacterial species using their biochemical, proteomic, and genomic properties (Fournier & Drancourt, 2015). This approach has the advantage of exhibiting a higher inter and intralaboratory reproducibility when compared to DNA‐DNA hybridization and chemotaxonomic methods. Based on MALDI‐TOF‐MS analysis, 16S rRNA gene sequence comparison (<95% similarity), genome comparison, AGIOS and dDDH values, we propose the creation of the new genus *Raoultibacter* gen. nov within the family *Eggerthellaceae* that belongs to the phylum Actinobacteria. Members of this family belong to the class Coriobacteriia. Many revisions have been made to the classification of this group by using various molecular techniques and Gupta et al. (2013 proposed the taxonomic division of this class into two orders (Coriobacteriales and Eggerthellales) and three families including *Coriobacteriaceae*,* Atopobiaceae*, and *Eggerthellaceae* (Stackebrandt, Rainey, & Ward‐Rainey, 1997). Members of the *Eggerthellaceae* are predominantly anaerobic, nonsporeforming, catalase and Gram‐positive, rods or cocci. As well, strains Marseille‐P2849^T^ and Marseille‐P3277^T^ are Gram‐positive. Most of the species closely related to the genus *Raoultibacter* gen. nov. were isolated from the human gut microbiota and, to date, exhibited a low pathogenicity (Gardiner et al., 2014; Lee et al., 2012).

## 5 CONCLUSION

The biochemical, proteomic, genetic, and genomic characteristics of strains Marseille‐P2849^T^ and Marseille‐P3277^T^ confirmed that they belong to two distinct species within a new genus in the family *Eggerthellaceae*, for which we propose the names *Raoultibacter* gen. nov., *R. massiliensis* sp. nov., and *R. timonensis* sp. nov. The type strain of *R. massiliensis* sp. nov., Marseille‐P2849^T^, was isolated from the feces of a 19‐year‐old healthy male Saudi Bedouin, whereas the type strain of *R. timonensis* sp. nov., Marseille‐P3277^T^, was isolated from the feces of a healthy 11‐year‐old Pygmy female living in Congo.

## 6 TAXONOMIC AND NOMENCLATURAL PROPOSALS

### 6.1 Description of *Raoultibacter* gen. nov.

*Raoultibacter (ra.ou.l.ti.bac'ter*. N.L. masc. n, “*Raoultibacter*,” composed of *Raoult*, in the honor of the French microbiologist Didier Raoult, founder of the IHU Mediterranée‐Infection in Marseille and inventor of culturomics, the culture strategy that has enabled the discovery of more than 250 bacterial species, and *bacter*, for rod).

*Raoultibacter* forms transparent microcolonies on blood agar with a mean diameter of 0.1–0.4 mm. Cells are Gram‐positive, nonsporeforming, motile rod that grow in microaerophilic and anaerobic atmospheres, with an optimal growth at 37°C after 48 hr of incubation. The pH tolerance ranges from 6 to 8.5. The type species of the genus is *R. massiliensis* sp. nov. The type strain of the genus is strain Marseille‐P2849^T^.

### 6.2 Description of *Raoultibacter massiliensis* sp. nov.

*Raoultibacter massiliensis* (*mas.si.li.en'sis*. L. masc. adj. *massiliensis*, from Massilia, the Latin name of Marseille, where the type strain was first isolated).

*Raoultibacter massiliensis* is a Gram‐positive and motile rod whose individual cells measure 0.8–1.2 µm in length and 0.4–0.6 µm in diameter. Transparent microcolonies obtained on 5% sheep blood‐enriched Columbia agar exhibit a diameter of 0.1–0.4 mm. The optimal growth is observed at 37°C after 48 hr of incubation. It is oxidase negative but catalase positive. Indole is produced. Using API strips, positive reactions are observed with glycerol, d‐ribose, d‐galactose, d‐glucose, d‐fructose, d‐mannose, d‐mannitol, *N*‐acetylglucosamine, amygdaline, arbutin, esculin ferric citrate, salicin, d‐maltose, d‐lactose, d‐saccharose, d‐trehalose, d‐melezitose, gentiobiose, d‐tagatose, potassium gluconate, l‐arabinose, gelatine, d‐cellobiose, d‐melezitose, d‐raffinose, d‐sorbitol, amidon, and potassium 5‐ketogluconate. Fucosidase, esterase (C4), esterase lipase (C8), lipase (C14), leucine arylamidase, valine arylamidase, cystine arylamidase, acid phosphatase, and naphthol phosphohydrolase activities are present but no reaction is obtained for urease and alkaline phosphatase. The major fatty acids are 9‐octadecenoic acid (36%), hexadecanoic acid (18%), and tetradecanoic acid (13%). The genome is 3,657,161 bp long with a DNA G+C content of 59.02 mol%. The 16S rRNA and genome sequences were both deposited in EMBL/EBI under accession numbers LT576395 and FZQX00000000, respectively. The habitat of this bacterium is the human gut. The type strain Marseille‐P2849^T^ (= CSUR P2849 = DSM 103407) was isolated from a stool specimen of a healthy 19‐year‐old male Bedouin living in Saudi Arabia.

### 6.3 Description of *Raoultibacter timonensis* sp. nov.

*Raoultibacter timonensis* (*ti.mo.nen'sis*, N.L. masc. adj., *timonensis* pertaining to La Timone, the name of the university hospital in Marseille, France, where the strain was first isolated).

*Raoultibacter timonensis* is a Gram‐positive and motile rod whose individual cells measure 1–2 µm in length and 0.35–0.44 µm in diameter. Transparent microcolonies grown on 5% sheep blood‐enriched Columbia agar have a diameter of 0.1–0.4 mm with an optimal growth at 37°C after a 48 hr incubation period in anaerobic conditions. It is oxidase negative and catalase positive. Using API strips, positive reactions are observed with glycerol, d‐ribose, d‐galactose, d‐glucose, d‐fructose, d‐mannose, d‐mannitol, *N*‐acetylglucosamine, amygdaline, arbutin, esculin ferric citrate, salicin, d‐maltose, d‐lactose, d‐saccharose, d‐trehalose, d‐melezitose, gentiobiose, d‐tagatose, methyl‐ αd‐glucosamine, d‐cellobiose, d‐turanose, l‐rhamnose, glycerol, potassium gluconate, l‐arabinose, gelatin, d‐cellobiose, d‐melezitose, d‐raffinose, and d‐sorbitol. Trypsin, α‐chymotrypsin, α‐galactosidase, β‐galactosidase, β‐glucuronidase, α‐glucosidase, β‐glucosidase, *N*‐acetyl‐β‐glucosaminidase, α‐mannosidase, exhibited esterase (C4), esterase lipase (C8), lipase (C14), leucine arylamidase, valine arylamidase, cystine arylamidase, acid phosphatase, and naphthol phosphohydrolase activities are present. No reactions are obtained for urease and phosphatase alkaline. The major fatty acids are 9‐octadecenoic acid (38%), hexadecanoic acid (25%), and tetradecanoic acid (11%). The genome is 4,000,215‐bp long with a DNA G+C content of 59.9 mol%. The 16S rRNA and genome sequences were deposited in EMBL/EBI under accession numbers LT623894 and OEPT00000000, respectively. The habitat of this bacterial strain is the human gut. The type strain Marseille‐P3277^T^ (= CSUR P3277 = CCUG 70680) was isolated from the human stool of a 11‐year‐old healthy Pygmy female.

## CONFLICT OF INTEREST

The authors declare no conflict of interest.

## AUTHORS CONTRIBUTION

Sory Ibrahima Traore and Melhem Bilen isolated the bacteria and drafted the manuscript. Sory Ibrahima Traore, Melhem Bilen, Maxime Descartes Mbogning Fonkou, and Fréderic Cadoret participated to experiment for phenotypic characterization of these strains. Caroline Michelle performed the genomic sequencing. Fadi Bittar contributed to phylogenic analysis. Mamadou Beye, Awa Diop, and Mamadou Lamine Tall contributed to bioinformatic analysis and drafted manuscript. Muhammad Yasir, Esam Ibraheem Azhar, Fehmida Bibi, Asif Ahmad Jiman‐Fatani, and Ziad Daoud provided the samples and edited the manuscript. Pierre‐Edouard Fournier reviewed the results and edited the manuscript. Sophie Edouard designed the study, supervised the project, wrote and finalized the manuscript.

## ETHICS STATEMENT

The donors gave a signed informed consent, and the study was validated by the ethics committee of the Institut Federatif de Recherche 48 under number 09‐022.

## Data Availability

The 16S rRNA and genome sequences were both deposited in EMBL/EBI under accession numbers LT576395 and FZQX00000000, respectively.
