# EXPRESSION OF CONCERN: Noncontiguous Finished Genome Sequence and Description of *Raoultibacter massiliensis* gen. nov., sp. nov. and *Raoultibacter timonensis* sp. nov, Two New Bacterial Species Isolated from the Human Gut

**DOI:** 10.1002/mbo3.70251

**Published:** 2026-02-19

## Abstract

**EXPRESSION OF CONCERN**: S.I. Traore, M. Bilen, M. Beye, A. Diop, M.D.M. Fonkou, M.L. Tall, C. Michelle, M. Yasir, E.I. Azhar, F. Bibi, F. Bittar, A.A. Jiman‐Fatani, Z. Daoud, F. Cadoret, P.‐E. Fournier, and S. Edouard, “Noncontiguous Finished Genome Sequence and Description of *Raoultibacter massiliensis* gen. nov., sp. nov. and *Raoultibacter timonensis* sp. nov, Two New Bacterial Species Isolated from the Human Gut,” *Microbiology Open* 8, no. 6 (2019): e00758, https://doi.org/10.1002/mbo3.758.

This Expression of Concern is for the above article, published online on 30 January 2019 in Wiley Online Library (wileyonlinelibrary.com), and has been issued by John Wiley & Sons Ltd. The Expression of Concern has been agreed due to questions raised about the study's adherence to French legal and ethical requirements for research involving human subjects, including questions regarding proper informed consent for research involving vulnerable populations. Additionally, a third party noted that the two bacterial strains reported in this article may have been previously reported by some of the same authors in different publications. The investigation into these concerns is ongoing. Therefore, the journal has decided to issue an Expression of Concern to inform and alert readers.

