# Supplementary figures and images for: Noncontiguous finished genome sequence and description of Raoultibacter massiliensis gen. nov., sp. nov. and Raoultibacter timonensis sp. nov, two new bacterial species isolated from the human gut
Source: Microbiologyopen. 2019 Jan 30;8(6):e00758. doi: 10.1002/mbo3.758 (PMC6562231; doi:10.1002/mbo3.758)

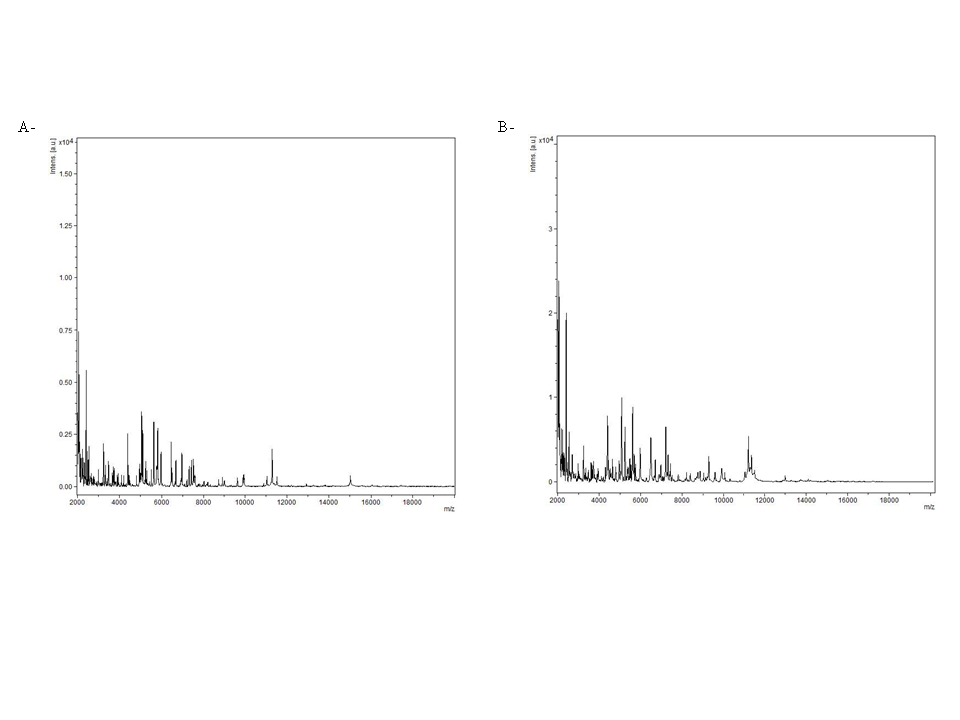

Supplement: Supplementary file 1 [file MBO3-8-e00758-s001.tif]
